# Numerical investigation of band gaps in 3D printed cantilever-in-mass metamaterials

**DOI:** 10.1038/srep28314

**Published:** 2016-06-22

**Authors:** Awais Qureshi, Bing Li, K. T. Tan

**Affiliations:** 1 Department of Mechanical Engineering, The University of Akron, Akron, OH 44325-3903. USA

## Abstract

In this research, the negative effective mass behavior of elastic/mechanical metamaterials is exhibited by a cantilever-in-mass structure as a proposed design for creating frequency stopping band gaps, based on local resonance of the internal structure. The mass-in-mass unit cell model is transformed into a cantilever-in-mass model using the Bernoulli-Euler beam theory. An analytical model of the cantilever-in-mass structure is derived and the effects of geometrical dimensions and material parameters to create frequency band gaps are examined. A two-dimensional finite element model is created to validate the analytical results, and excellent agreement is achieved. The analytical model establishes an easily tunable metamaterial design to realize wave attenuation based on locally resonant frequency. To demonstrate feasibility for 3D printing, the analytical model is employed to design and fabricate 3D printable mechanical metamaterial. A three-dimensional numerical experiment is performed using COMSOL Multiphysics to validate the wave attenuation performance. Results show that the cantilever-in-mass metamaterial is capable of mitigating stress waves at the desired resonance frequency. Our study successfully presents the use of one constituent material to create a 3D printed cantilever-in-mass metamaterial with negative effective mass density for stress wave mitigation purposes.

Advancement made to the world of materials over the past few decades has brought on an era of uniquely engineered materials. The highest demand for performance of materials coupled by the fast pace of scientific developments have pushed boundaries beyond the imagination of man to design and engineer materials with exceptional properties not found in nature. An increasing amount of research has been conducted to explore the extraordinary physical effective properties (like negative effective mass density, negative effective elastic modulus and negative Poisson’s ratio, etc) of acoustic/elastic/mechanical metamaterials^1^,^2^,^3^,^4^,^5^. Such properties are often realized through the fabrication of specifically designed structures at the meso-, micro- and nano-scale, and not by material chemical composition. The potential applications of these unique metamaterials range from vibration isolation^6^,^7^,^8^, acoustic sound wave control^9^,^10^,^11^, impact and blast-wave mitigation^12^,^13^,^14^, etc. The wave attenuation and mitigation mechanism exhibited by the mechanical metamaterials is a result of the formation of frequency band gaps that prohibited transmission of acoustic/mechanical waves^15^,^16^,^17^,^18^,^19^.

The control over band gaps in the negative effective mass concept employs a mass-in-mass model which defines a unit cell with an outer mass and an inner mass connected by a spring^20^,^21^,^22^. The system exhibits the dynamic negative mass behavior due to the internal resonance of the unit cell caused by the inner mass and the spring, as depicted in Fig. 1(a). The practical realization of these mass-in-mass metamaterials remains a challenge, as they either represent cumbersome structures^13^,^22^, or require more than one constituents for manufacturing (for example, heavy core as inner mass and surrounded by soft elastic layer, acting as spring)^1^,^23^, which does not make fabrication easy, particularly if mass production of numerous unit cells is required in the entire structure.

The frequency band gaps of a metamaterial can also be controlled using phononic plates which employ beam-like resonators to induce local resonance^24^,^25^. Other studies show wave attenuation in thin metamaterial plates as well as enhancements by introducing springboards^26^. The plates are created using a cantilever-mass-microstructure, to analyze the dispersion relations for in-plane longitudinal waves^27^. Each design has proven to be effective at impact wave mitigation through the presence of band gaps, where the input frequency falls within the locally resonant band gap frequency of the internal structure. However, there is a lack of design parameters to govern the designed resonance frequency based on analytical model of cantilever-in-mass metamaterials. Moreover, the prototypes in earlier studies were typically made of metals^25^,^27^, thus quick and easy fabrication could not be simply achieved.

Recent progress in three-dimensional (3D) printing technology has enabled a rapid prototyping of complex models at a limited cost. Fabrication of metamaterials with complex internal architecture is a challenge in traditional manufacturing methodology. However, 3D printing offers possible realization of mechanical metamaterials, since in 3D printing, complex structural shapes and geometries can be achieved, without incurring assemblage and mold requirement, and thus reducing cost and increasing efficiency in fabrication^28^. Another manufacturing approach of elastic metamaterial is by the precision laser cutting system as demonstrated by Zhu *et al*.^29^.

Early research conducted on the rapid prototyping of band gap microstructures revealed that it is possible to use modern 3D printing techniques to create features at a micro level. The outcome exhibits the formation of a frequency band gap in the material^30^. In the effort to 3D print a mass-in-mass metamaterial with spring-connected mass, Buckmann *et al*.^31^ fabricated the delicate helical Hooke’s spring structures combined with the inner cube’s solid material, but realized that the springs are not smooth and have slightly larger than designed average wire diameter. Although 3D printing provides a lot of flexibility in design, it is still difficult to 3D print springs accurately. As such, there appears a need to reconsider the use of mass-in-mass metamaterial model, and possibly consider the better use of a cantilever-in-mass metamaterial design in 3D printing. The lack of analytical model based design and the need to achieve a one constituent material tunable mechanical metamaterial to enable easy fabrication by 3D printing are the main motivations for this work.

In this work, we propose the design, fabrication and characterization of 3D printable mechanical metamaterials by employing cantilever beam-like resonators to equivalently and effectively represent the spring mass-in-mass model. We develop an analytical model of analogous cantilever-in-mass metamaterial based on Bernoulli-Euler beam theory. We perform parametric studies to understand and evaluate the influence of geometrical and material properties on the designed resonance bandgap of the metamaterials, with the aim to achieve tunability. We further validate the analytical model using 2D finite element simulation and investigate the wave attenuation performance of the metamaterial. Finally, we fabricate and demonstrate 3D printable metamaterials using Objet Eden260v 3D printer. We also conduct numerical experiments of the 3D printable metamaterials and validate the 2D simulation results and analytical model to confirm the feasibility of the design.

## Results

### Mass-in-mass model

**Figure 1:**
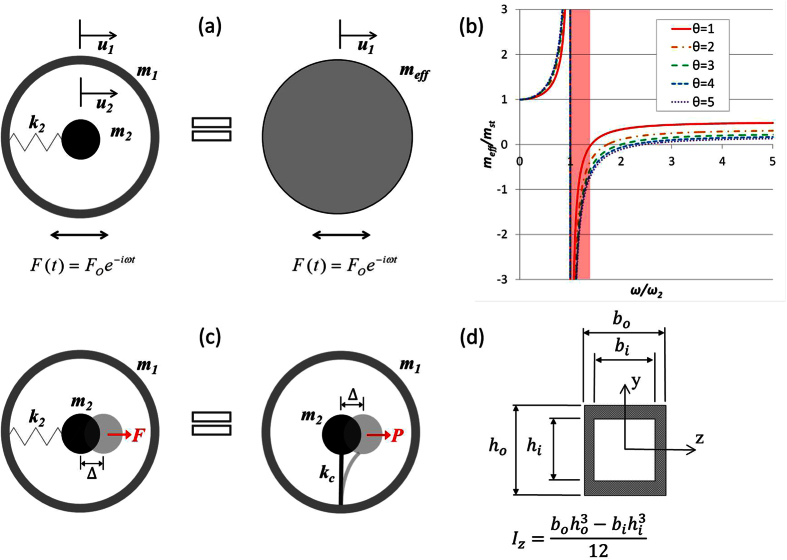
(**a**) Mass-in-mass unit cell and its equivalent effective mass model. (**b**) Plot showing normalized mass against normalized frequency and highlighting the negative mass density region for *θ* = 1. (**c**) Equivalency of a mass-in-mass unit cell with a cantilever-in-mass unit cell. (**d**) Calculating moment of inertia of a rectangular beam.

This section briefly reviews the theory of negative effective mass density obtained by using a mass-in-mass spring system^20^,^21^. Such a system would consist of an outer mass, *m*_1_, and an inner mass, *m*_2_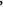 connected by a spring of stiffness *k*_2_, as illustrated in Fig. 1(a). Assuming that the external applied force *F* and masses displacement *u*_*γ*_ (γ = 1, 2) are governed by harmonic motion, as in Equations (1) and (2), we can use the harmonic wave behavior to derive equations in terms of the applied force.


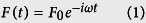



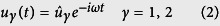


Applying Newton’s second law and free body diagram for each of the masses, we obtain the expressions for the forces acting on the mass *m*_1_ and *m*_2_.

Outer mass, *m*_1_:


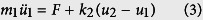


Inner mass, *m*_2_:


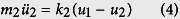


Solving the above equations using the harmonic wave behavior, we simplify to


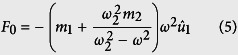


where 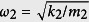 is the locally resonant frequency of the internal resonator.

We equate the motion of the outer mass to that of an effective mass, 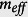, and use the free body diagram to derive an expression for the forces acting on the effective mass of the system.


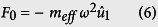


Comparing Equations (5 and 6) yields the effective mass, 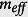, in terms of the locally resonant frequency of the structure, *ω*_2_ and the input frequency, *ω*.


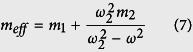


From Equation (7), we see that as the input frequency *ω* gets close to locally resonant frequency *ω*_2_, there is a vertical asymptote which approaches positive infinity from one side and negative infinity from the other. Within a certain range 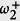 we observe a region where the effective mass of the system exhibits negative behavior, which is also the region where we see the formation of a frequency band gap.

Considering the stationary mass of the system as *m*_*st*_ = *m*_1_ + *m*_2_, we derive the normalized mass 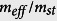 as a function of the normalized operating frequency 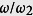. Simplifying Equation (7), we have


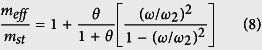


We can see from Equation (8) that the normalized mass is a function of the normalized frequency 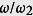 and the term *θ* which is defined as the ratio of the inner mass to the outer mass, 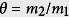. Plotting the normalized mass against the normalized operating frequency, we observe the negative effective mass of the unit cell as presented in Fig. 1(b). The shaded region highlights the frequency domain where the effective mass is negative for *θ* = 1. We can see from Fig. 1(b) the effect of varying *θ* on the negative mass region. As the ratio of the inner mass to the outer mass increases, the frequency range for the negative effective mass of the unit cell also increases. For *θ* = 1 used in this study, the negative effective mass is in the region of *ω*_2_ < *ω* < 1.3*ω*_2_.

### Cantilever-in-mass model

We transform the spring mass-in-mass model into a cantilever-in-mass model to observe the similar negative effective mass phenomenon. Since elastic structures behave like springs‒deflecting when a force is applied, the stiffness of a beam can be defined in the same way as for a spring. In the model depicted in Fig. 1(c), we use Bernoulli-Euler beam theory to find the deflection of the beam and its equivalent spring constant where Δ is the deflection in the spring or beam.

Linear spring:


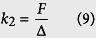


Cantilever beam:


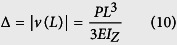


where *v*(*x*) is the transverse displacement function of beam length *L*, and *P* is the applied load at the end of the beam (*x* = *L*).


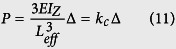



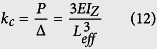


The “spring constant”, *k*_c_ of a cantilever beam is derived in Equation (12), where 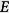 is the Young’s Modulus of the beam, *I*_*z*_ is the moment of inertia about the bending z-axis [formula given in Fig. 1(d) for a rectangular cross section], and *L*_*eff*_ is the effective length of the beam. The equivalency of a mass-in-mass unit cell with a cantilever-in-mass unit cell, 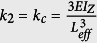 is schematically presented in Fig. 1(c). Using the relationship 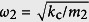 and Equation (12), we can attain the resonance frequency of the cantilever-in-mass model as.


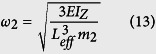


**Figure 2:**
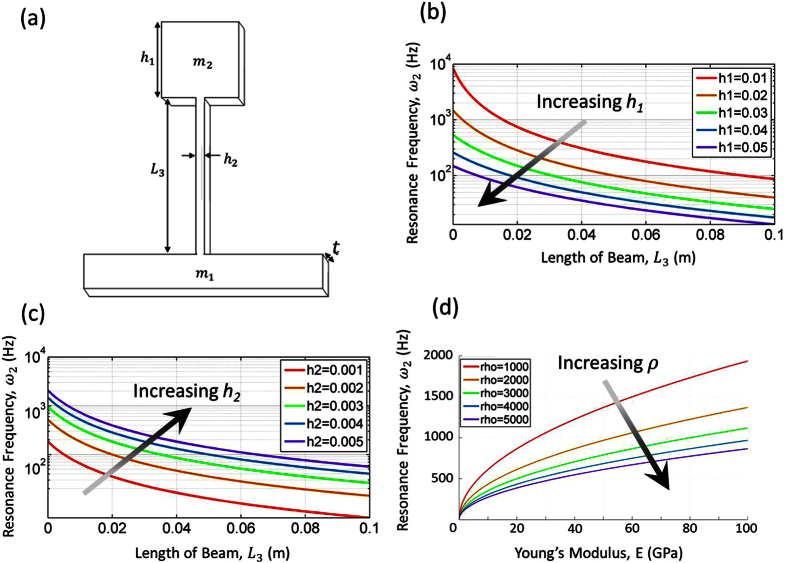
(**a**) Cantilever-in-mass practical design with important geometrical parameters. Variation of designed resonance frequency with geometrical changes in cantilever-in-mass structure (**b**) length of beam, *L*_3_ and dimension of mass, *h*_1_ (**c**) length of beam, *L*_3_ and width of beam, *h*_2_. (**d**) Variation of designed resonance frequency with changes in material mass density, 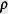 and Young’s Modulus, E.

In the practical design displayed in Fig. 2(a), the resonator mass *m*_2_ is connected to a cantilever beam of length *L*_3_, width *h*_2_ and thickness *t*. In order to simplify the approach, we assume the resonator mass to be a square defined by the dimension *h*_1_. Given all the dimensions and assuming a one constituent material with density *ρ*, we have


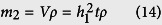



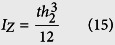



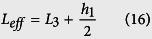


We substitute Equations (14, 15, 16) into Equation (13) to obtain the resonance frequency in terms of the design parameters. It is important to note that the effective length of the beam *L*_*eff*_ is considered from the base to the center of the resonating mass, defined by Equation (16). This assumption was validated during parametric numerical study by varying beam length, and comparing the resonance frequency evaluated numerically with that from the analytical model. As such, the resonance frequency of the cantilever-in-mass model with geometrical and material parameters is given by


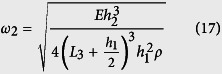


It is interesting to note that due to the moment of inertia of the beam, *I*_z_ and the resonator mass, *m*_2_, the resonance frequency of the cantilever-in-mass model is independent of thickness, *t*.

### Factors influencing locally resonant frequency

Equation (17) can be expressed by the following notation showing the dependence of the resonance frequency on five variables.


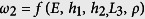


These variables are considered and analyzed into two categories: geometric dimensions and material properties. We investigate how the geometric parameters and the material selection influence the locally resonant frequency of the structure in Fig. 2(b–d). We can observe from Fig. 2(b) that as the length of the beam increases, the resonance frequency decreases. Coupling with the effect of mass dimension, we see that as we increase *h*_1_, there is a further decrease in the resonance frequency of the structure. This is directly correlated to the decrease in resonance frequency caused by the overall length of the beam, as the increase in dimension of the mass also increases the effective length of the beam, *L*_*eff*_. In contrast, when you increase the width of the beam, *h*_2_ there is an increase in the resonance frequency, as shown in Fig. 2(c). Figure 2(b,c) demonstrate how the resonance frequency can be tuned and adjusted according to the geometrical parameters (*h*_1_, *h*_2_, *L*_3_).

We also analyze the influence of material properties (*E*, *ρ*) on the designed resonance frequency. Figure 2(d) illustrates the effects of varying the Young’s modulus, 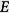 and the material density, *ρ*. It is evident that as Young’s modulus increases, the resonance frequency increases as well. However, increasing the material density will decrease the resonance frequency of the cantilever-in-mass metamaterial. The material properties, influenced by the selection of materials, are particularly important in this work, as the material chosen must be able to be used in the 3D printer Objet Eden260v. The general application of the cantilever-in-mass analytical model can, however, be applied to any types of materials.

### Band gap by frequency sweep

**Figure 3:**
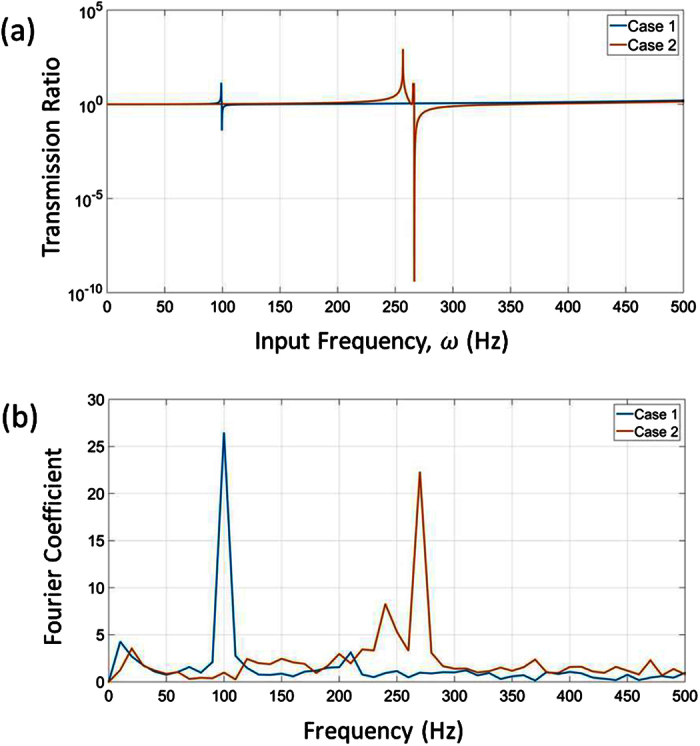
(**a**) Frequency sweep across the 2D model for case 1 and case 2 at designed resonance frequency of 100 Hz and 277 Hz, respectively. (**b**) Fast Fourier Transformation of the first cantilever-in-mass resonator for case 1 and case 2.

A two-dimensional (2D) finite element (FE) model of the cantilever-in-mass structure was created in COMSOL Multiphysics to validate the derived analytical model. Further details of the 2D FE model should be referred to Methods Section. The frequency sweep result of the 2D FE model is shown in Fig. 3(a), comparing the design case 1 and case 2 (Table 1). In both of the designs we can see dips in the transmission ratio. The blue curve shows the frequency sweep for case 1 and a dip is observed at *ω* = 100 *Hz*. The red curve represents the frequency sweep for case 2 with a dip evident at *ω* = 277 *Hz*. In both cases, the results from the FE simulation have very good agreement with the designed resonance frequency predicted by the cantilever-in-mass analytical model Equation (17).

This dip in displacement ratio proves that there is energy reduction as the wave propagates through the metamaterial. According to the negative effective mass model explained in earlier section, as 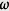 approaches the locally resonant frequency *ω*_2_ of the structure, the effective mass approaches positive infinity from one side and negative infinity from the other side. This phenomenon is clearly exhibited in the frequency sweep simulation.

It is worth noting that the band gap region for negative mass starts from the dip at the designed resonance frequency and ends when the transmission ratio returns to 1. Case 1 shows a narrower band gap region compare to Case 2. This is expected as the negative effective mass region is dependent on the locally resonant frequency (the region is *ω*_2_ < *ω* < 1.3*ω*_2_ for *θ* = 1 used in this study), so higher resonance frequency naturally has broader band gap region.

### Fast Fourier Transform of internal resonator

Figure 3(b) displays the Fast Fourier Transform (FFT) of the first resonator in both design case 1 and case 2. FFT plot provides clear understanding of the vibrational frequency and energy absorption of the internal mass during wave propagation through the metamaterial. As defined by the negative effective mass of the analytical cantilever-in-mass model, the internal resonance of the metamaterial is caused by the beam and the resonating internal mass. FFT results presented in Fig. 3(b) clearly show that the resonance peaks of the internal mass correspond in good agreement with the frequency dips seen in frequency sweep results [Fig. 3(a)]. This means that the internal resonators are absorbing the wave propagation energy at their respective designed resonance frequency.

### Wave attenuation at band gap region

**Figure 4:**
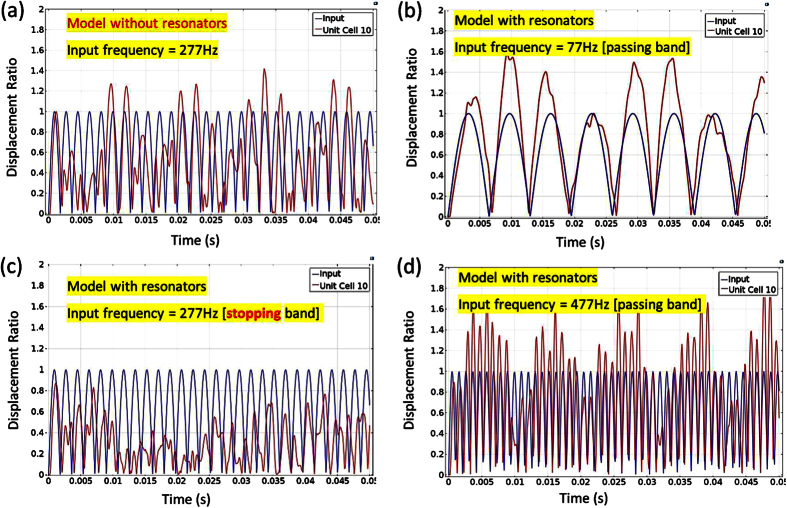
(**a**) Displacement profile of the 10^th^ unit cell for model with no resonators at input frequency of 277 Hz. Displacement profile of the 10^th^ unit cell for model with resonators at input frequency of (**b**) 77 Hz [passing band], (**c**) 277 Hz [stopping band gap], and (**d**) 477 Hz [passing band].

The locally resonant frequency of the cantilever-in-mass structure results in the presence of frequency band gaps at the designed frequency region. As introduced in the mass-in-mass model for negative effective mass, when the input frequency approaches the locally resonant frequency, the negative effective mass behavior is exhibited and this causes wave attenuation at the designed frequency. In order to observe wave attenuation at specific frequency input, we plot and compare the displacement of the 10^th^ unit cell (output) and 1^st^ unit cell (input). Figure 4(a) shows the result of the model without resonators (not a cantilever-in-mass metamaterial). It is clear that stress wave propagates through the material without any attenuation. For the model with resonators (cantilever-in-mass metamaterial with designed frequency at 277 Hz), three different harmonic input frequencies (according to Equation 18) are given for different cases. We can see that when the input frequency is within the passing band of the metamaterial [77 Hz and 477 Hz shown in Fig. 4(b–d)], waves propagate through the metamaterial without attenuation. However, when the input frequency falls within the stopping band gap of the cantilever-in-mass structure [277 Hz in Fig. 4(c)], significant wave attenuation is clearly observed.

For frequencies which are outside the band gap (passing band), we can see that the displacement at the 10^th^ unit cell is larger than the input. This is due to the reflection and interaction of the propagating stress waves. At passing band input frequency of 77 Hz, we observe a 24.8% increase in the average displacement. At 477 Hz input frequency, we notice a 16.5% increase in the average displacement of the unit cell. However, when the input frequency is within the stopping band region at 277 Hz, a huge reduction of 52.6% in the average displacement of the 10^th^ unit cell is achieved.

### Band gap for 3D numerical experiment

**Figure 5:**
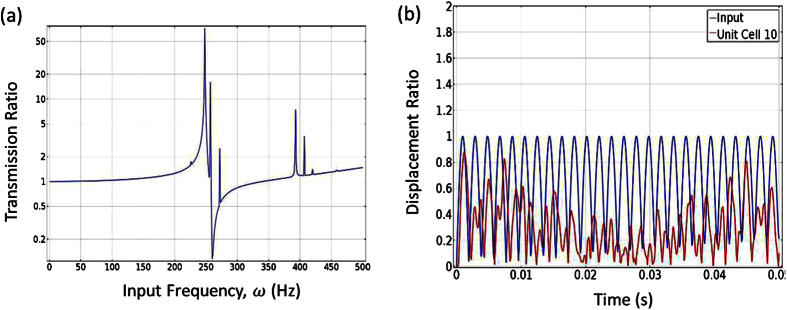
(**a**) Frequency sweep across the 3D model for designed resonance frequency of 277 Hz. (**b**) Displacement profile of the 10^th^ unit cell at input frequency of 277 Hz [stopping band gap].

According to the analytical model, the cantilever-in-mass structure should be independent of thickness. In order to validate this hypothesis, a 3D FE model was created and simulated. The 2D model was given a thickness of 1 centimeter to create the 3D model. A frequency sweep was performed from 0 to 500 Hz. Figure 5(a) reveals the transmission ratio at the 10^th^ unit cell across the frequency sweep. From the data gathered by the FE analysis, we observe a band gap forming around 260–350 Hz, resembling the frequency sweep result for the 2D model [Fig. 3(a)]. In order to validate wave attenuation performance, we plot the displacement profiles of 1^st^ unit cell (input) and 10^th^ unit cell (output) when the input frequency is around 277 Hz [Fig. 5(b)]. It is evident that significant wave attenuation occurs. Again the result obtained is very similar to the results from the 2D FE analysis [Fig. 4(c)]. The slight difference (of ~6%) in the frequency dip can be considered negligible and be attributed to the change in mesh size from the 2D model to a 3D model. The data acquired from the 3D simulation validates the assumption that the cantilever-in-mass analytical model is indeed independent of thickness, as well as provide numerical experiment demonstration for the cantilever-in-mass structure.

### Realization of 3D printed metamaterials

**Figure 6:**
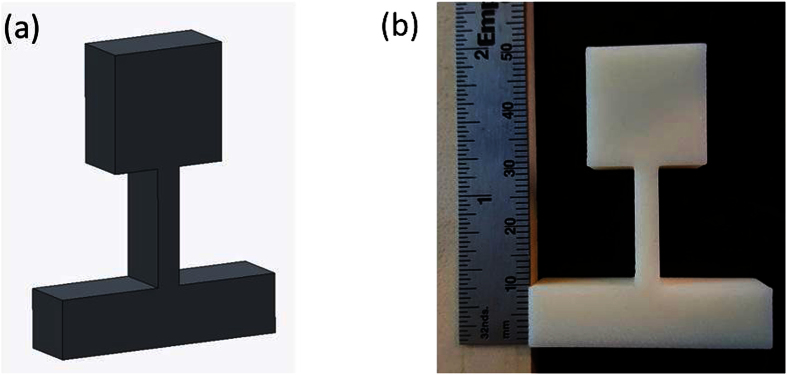
3D model and realization of cantilever-in-mass unit cell (**a**) CAD model. (**b**) Prototype of 3D printed part.

The Objet Eden260v 3D printer was used to employ the photopolymerization technique to 3D print a prototype of the cantilever-in-mass structure using single material (one material constituent). Figure 6(a,b) present the Computer-Aided Design (CAD) model and the prototype of the actual 3D printed part, thus demonstrating the realization of 3D printed cantilever-in-mass mechanical metamaterial. The authors plan to verify the analytical model of the cantilever-in-mass system using experimental data in the next phase of their research study. This will then provide a better understanding of the behavior of the structure in a practical scenario. The use of 3D printing and rapid prototyping will allow us to easily fabricate prototypes with different parameters and materials. We can switch from stiffer polymers such as VeroWhitePlus to more rubbery materials with very low Young’s modulus such as the TangoBlackPlus (*E* = 0.5*MPa*). 3D printing provides a valuable opportunity to explore a vast range of methods to fabricate 3D printable cantilever-in-mass mechanical metamaterial.

## Discussion

In this work, we have derived the analytical model of a mechanical metamaterial by converting the mass-in-mass model into a cantilever-in-mass model using the Bernoulli-Euler beam theory. The analytical model described by the parameters of the cantilever-in-mass structure demonstrated tunability of the designed resonance frequency by the influence of both geometrical dimensions and material parameters. A two-dimensional finite element model is created to validate the analytical results. Frequency sweep results confirm the frequency band gap as predicted by the analytical model. The negative effective mass behavior of the metamaterial is also exhibited by significant wave attenuation when input frequency is within the stopping band region of the designed resonance frequency. When incoming waves are at frequencies of the passing band, no wave attenuation is observed.

To demonstrate application and feasibility for 3D printing, the cantilever-in-mass model is further employed to design and fabricate 3D printable mechanical metamaterial. A three-dimensional numerical experiment is performed using COMSOL Multiphysics to validate the wave attenuation performance. Results show that the cantilever-in-mass metamaterial is capable of mitigating stress waves at the desired resonance frequency. This study successfully presents the use of one constituent material to create a 3D printed cantilever-in-mass metamaterial with negative effective mass density for stress wave mitigation purposes.

The flexibility of the cantilever-in-mass model will enable the design to be easily tuned and to achieve many different targeted resonance frequencies. In such a simply tunable model, it is more cost efficient to employ the use of 3D printing for the manufacturing of the cantilever-in-mass structure. With this control over the behavior of the metamaterial, we can design certain microstructures for other specific applications.

The authors believe that it is feasible to perform experimental testing on the 3D printed metamaterials. The input excitation can be created by an electro-dynamic shaker, and the output response of the metamaterial can be measured using Scanning Laser Doppler Vibrometry (SLDV) techniques to obtain the displacement and transmission ratio. The authors expect experimental testing data to agree well with numerical simulation results. However, the focus of this work is to derive an analytical model for the cantilever-in-mass metamaterial design, investigate its band gap tunability, demonstrate its 3D printability, and validate the design and performance using numerical experiment. The authors hope to demonstrate experimental verification in the near future and share their results with the scientific research community.

It is worth noting that the cantilever-in-mass structure presented in this manuscript is the simplest and most fundamental design possible. Its complexity can be significantly increased by designing a cantilever head that best utilizes and optimizes the space within the outer mass, yet allowing enough room and freedom to vibrate according to specific application. Moreover, complexity of the structure can be enhanced by using multi-materials, and 3D printing allows printing of multi-materials, such that it permits specific tuning of material properties for both the cantilever beam and resonator mass. Manufacturing using conventional technique might be cheaper under mass production, but specific tailoring of material structure and properties, and fabrication of small number of complex parts is more economical by using 3D printing. It is true that non-metal design has its disadvantage in load bearing and reliability. Depending on application, one option is to add a stronger material (metal) as a sacrificial plate when there is direct impact loading of high stress amplitude. Alternatively, one can use a relatively tough polymer that could be sufficient for load bearing purposes. The user has to make the decision on material selection based on design condition and application purposes. Nonetheless, this work proposes the analytical model for cantilever-in-mass model which could be employed for any materials.

## Methods

### Two-dimensional (2D) finite element model

A two-dimensional (2D) finite element (FE) model of the cantilever-in-mass structure was created in COMSOL Multiphysics to validate the derived analytical model and to prove the assumption that the resonance frequency is independent of thickness (Equation 17). We introduced two cases for the cantilever-in-mass structure, one at designed resonance frequency of 100 Hz and the other at 277 Hz (Table 1). The aim is to validate each design by comparing the designed resonance frequency (based on analytical model) and the dynamic wave attenuation performance of the system (based on FE simulation). The design parameters (listed in Table 1) were selected based on manufacturing feasibility and reasonable dimensional tolerance using 3D printing techniques. The material chosen in the FE simulation was VeroWhitePlus, which is a material type used in Objet Eden260v 3D printer, and was subsequently fabricated by 3D printing. The material properties for VeroWhitePlus were taken from the data sheet provided (*E* = 2,500 *MPa*, *v* = 0.33, *ρ* = 1,170 *kg*/*m*^3^).

**Figure 7:**
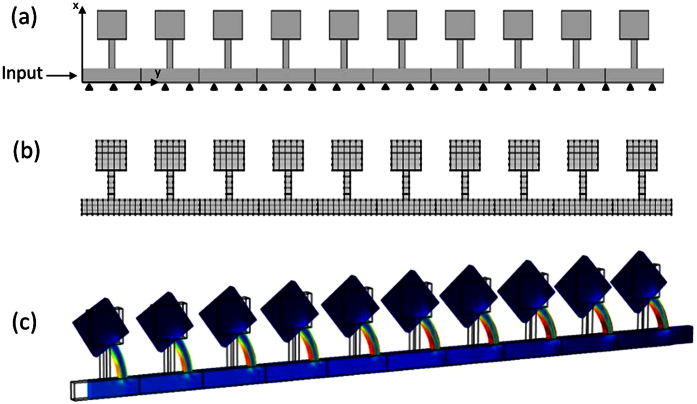
Numerical 2D model of the cantilever-in-mass system for Finite Element Analysis, showing 10 unit cells out of total 50 (**a**) Input and boundary conditions, (**b**) FE mesh. (**c**) 3D FE simulation of cantilever-in-mass model during stress wave propagation.

A FE model was created for each design to find the frequency band gap using frequency sweep analysis across the model. The geometry for a unit cell was created in COMSOL Multiphysics and was set to an array of 50 unit cells as shown in Fig. 7(a). The boundary condition represented constraint of the base in the *y*-direction, with the input given in the *x*-direction at the first unit cell. Analysis of wave propagation through the model was performed. Figure 7(b) portrayed the finite element mesh of the model.

We provided an input and performed a frequency sweep function (from 0 to 500 Hz) to observe any band gap. The transmission ratio, defined by the ratio of the displacement at the 10th unit cell (output) over the displacement of the 1st unit cell (input), was analyzed. In order to confirm the presence of a band gap, a harmonic excitation was further applied at specific frequency input using Equation (18), where *ω* is the input frequency in *hertz*, with the aim to observe wave attenuation at band gap frequency.


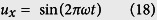


### Three-dimensional (3D) finite element model

In order to validate that the analytical model for the designed resonance frequency of the cantilever-in-mass metamaterial is independent of its thickness, a 3D FE model was created with a thickness of *t* = 0.01 *m*. Other than having a tangible thickness, the 3D FE model resembles that of the 2D FE model with the same material properties, geometrical dimensions, boundary and input conditions. The 3D FE simulation of the model during stress wave propagation is shown in Fig. 7(c). Results were compared to that of the 2D FE model. The data obtained were used to validate the assumption that the cantilever-in-mass model is independent of thickness. The 3D FE model was created similarly using COMSOL Multiphysics 5.0. More importantly, the 3D FE model represented a numerical experiment that allowed us to use 3D printing to validate our analytical and computational results by gathering experimental data in the next phase of our study.

### Fabrication using 3D printing

Based on the same design dimension as the 3D FE model, the feasibility of the design is demonstrated by 3D printing using the Objet Eden260v 3D printer, Stratasys Inc. The Eden260v is a commercialized 3D printer which makes use of the photopolymerization process to build a structure layer by layer. It makes use of photocurable polymers such as VeroWhitePlus which are sprayed by a nozzle onto the build path before being cured using ultra violet light.

## Additional Information

**How to cite this article**: Qureshi, A. *et al*. Numerical investigation of band gaps in 3D printed cantilever-in-mass metamaterials. *Sci. Rep.* **6**, 28314; doi: 10.1038/srep28314 (2016).

## Figures and Tables

**Table 1:** Design matrix used in FE numerical simulation.

Geometrical Parameters	Case 1	Case 2
* L*_3_ (m)	0.02	0.02
* h*_1_ (m)	0.02	0.02
* h*_2_ (m)	0.002	0.004
Designed Resonance frequency		
* ω*_2_ (Hz)	100	277
